# Fruitflow^®^: the first European Food Safety Authority-approved natural cardio-protective functional ingredient

**DOI:** 10.1007/s00394-016-1265-2

**Published:** 2016-07-07

**Authors:** Niamh O’Kennedy, Daniel Raederstorff, Asim K. Duttaroy

**Affiliations:** 1 Provexis PLC, Reading, UK; 2 Rowett Institute of Nutrition and Health, University of Aberdeen, Aberdeen, UK; 3 DSM Nutritional Products Ltd, 4002 Basel, Switzerland; 4 Department of Nutrition, Institute for Basic Medical Sciences, Faculty of Medicine, University of Oslo, POB 1046, Blindern, 0316 Oslo, Norway

**Keywords:** Tomato, Water-soluble tomato extract, Fruitflow^®^, Human platelets, Platelet activation, Polyphenols, ADP, Blood pressure, Angiotensin-converting enzyme, EU regulation 1924/2006, EFSA health claim

## Abstract

Hyperactive platelets, in addition to their roles in thrombosis, are also important mediators of atherogenesis. Antiplatelet drugs are not suitable for use where risk of a cardiovascular event is relatively low. It is therefore important to find alternative safe antiplatelet inhibitors for the vulnerable population who has hyperactive platelets in order to reduce the risk of cardiovascular disease. Potent antiplatelet factors were identified in water-soluble tomato extract (Fruitflow^®^), which significantly inhibited platelet aggregation. Human volunteer studies demonstrated the potency and bioavailability of active compounds in Fruitflow^®^. Fruitflow^®^ became the first product in Europe to obtain an approved, proprietary health claim under Article 13(5) of the European Health Claims Regulation 1924/2006 on nutrition and health claims made on foods. Fruitflow^®^ is now commercially available in different countries worldwide. In addition to its reduction in platelet reactivity, Fruitflow^®^ contains anti-angiotensin-converting enzyme and anti-inflammatory factors, making it an effective and natural cardio-protective functional food.

## Introduction

During the last 50 years, tomato (*Lycopersicon esculentum*) has become a highly consumed healthy food [1]. Tomato contains several components that are beneficial to overall health, including vitamin E, flavonoids, phytosterols, carotenoids, several water-soluble vitamins and minerals [2]. The presence of a variety of antioxidants such as polyphenols, e.g. quercetin, kaempferol, naringenin, and carotenoids, in considerable amounts makes tomato a rich source of antioxidants [3, 4]. Since oxidative stress triggers inflammatory disorders, the basis for the development of several diseases such as immune disorders, atherosclerotic lesions and rupture of plaque [5], antioxidants present in tomato are therefore believed to slow the progression of many diseases including cardiovascular disease (CVD). In order for tomato to provide cardio-protection, it must include bioactive factors that are able to reduce several cardiovascular risk factors such as LDL cholesterol, triglycerides, homocysteine, platelet hyperactivity and blood pressure [6].

**Fig. 1:**
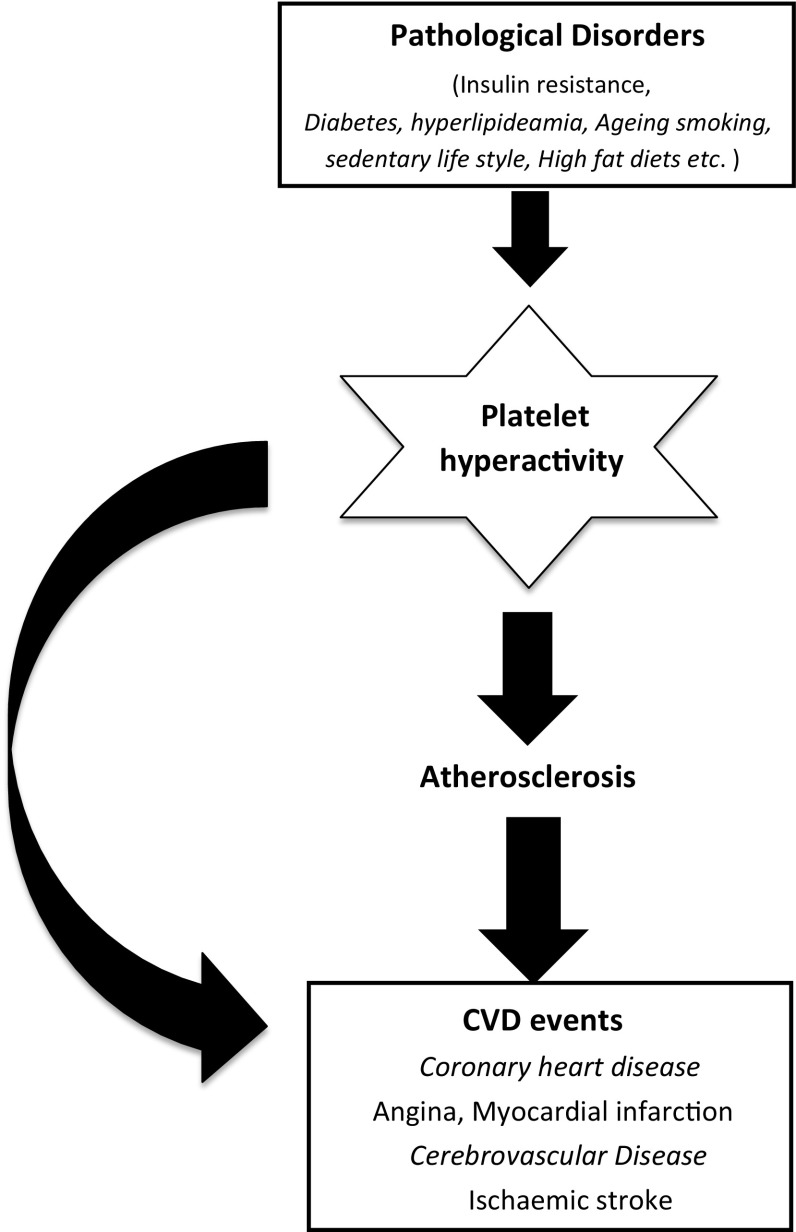
Hyperactivity of platelets and impact on health. Platelets play an important role in CVD both in the pathogenesis of atherosclerosis and in the development of acute thrombotic events. Hyperactive platelets are involved in the development of atherosclerosis by different mechanisms such as membrane shedding, growth factor secretion and expression of several adhesive factors. In addition, hyperactive platelets are involved in the well-known penultimate thrombotic events

Platelets play an important role in CVD both in the pathogenesis of atherosclerosis and in the development of acute thrombotic events (Fig. 1). Their importance in CVD is indirectly confirmed by the benefit of antiplatelet agents such as aspirin, clopidogrel and glycoprotein IIb/IIIa inhibitors abciximab/eptifibatide [7]. In fact, intravascular thrombosis is a factor in the generation of a wide variety of CVDs. Platelets in individuals with diabetes, sedentary lifestyle, obesity and insulin resistance show increased activity at baseline and in response to agonists, ultimately leading to increased aggregation and plaque development [8–10]. Aspirin remains a cornerstone of antiplatelet therapy but does not benefit all patients equally, as evidenced by the phenomenon of aspirin resistance [7]. Aspirin therapy is also responsible for a number of serious side effects, rendering it unsuitable for use in primary prevention of CVD [11, 12]. However, very few new antithrombotics are currently progressing beyond phase II trials, and those that have been developed are similarly unsuitable for use in primary prevention [12]. There is an interest in naturally occurring compounds which might lack the side effects currently so prevalent. We therefore systematically investigated the effects of bioactive compounds in fruits and vegetables on human blood platelet aggregation and utilized the findings to characterize the mechanisms involved in this process, in the hope of identifying potential dietary antiplatelet components. In a variety of studies, it was demonstrated that water-soluble components of tomatoes are capable of inhibiting platelet aggregation both in vitro and in vivo [13–16]. These water-soluble tomato components were also found to inhibit angiotensin-converting enzyme (ACE) and to relax the vascular endothelium, the other important limbs of the cardiovascular system [17, 18]. A water-soluble tomato extract containing all the bioactive components was developed and later given the trade name Fruitflow^®^. Fruitflow^®^ is now an established naturally derived functional food ingredient, marketed globally. Since its discovery in 1999, several mechanistic studies and human trials with Fruitflow^®^ have been carried out. Studies included localization of the antiplatelet activity within the tomato fruit, its modes of action, its stability under various conditions and identification of the compounds with antiplatelet activity. The presence of a range of compounds suggested that all have antiplatelet activity but act on different parts of the platelet activation/aggregation pathway. The chemical properties of the active compounds indicated their potential suitability as therapeutic agents or as functional food ingredients. There are several excellent reviews available on overall health benefits of tomatoes [1, 2, 4, 5, 19, 20]. This review will discuss the background to Fruitflow^®^ discovery and the body of biological and regulatory work involved in the granting of its authorized health claim in Europe and acceptance as a functional food by regulatory authorities worldwide.

## Epidemiology of tomato consumption and CVD risk reduction

Epidemiologic studies focused on tomato and tomato products associated their intake with a reduced risk of CVD [19, 21, 22]. However, tomatoes and tomato-based products are important dietary sources of lycopene in observational studies, and most human lycopene trials are performed using tomato-based interventions. Studies showed that increased plasma lycopene levels were associated with reductions in CVD risk factors [23–25]. The strongest population-based evidence for the beneficial effects of tomato lycopene came from a multi-centre case–control study [26]. However, a subsequent dietary intervention study observed that consumption of a carotenoid-rich diet did not have an effect on plasma antioxidant status or markers of oxidative stress [27]. Several [25, 28], but not all [28–32] prospective studies relating circulating lycopene concentrations and CVD risk have reported inverse associations, while studies based on dietary intake did not find any such significant associations [32–36]. Also, the focus on lycopene in tomatoes cannot explain the fact that, taking tomato consumption into account, individuals in the Mediterranean area have a lower risk of CVD when compared to their North American and other European counterparts [2, 37, 38]. Thus, it is difficult to separate out the potential lycopene contribution to cardiovascular health from the overall contribution from tomato products and other components present in tomatoes. The lack of coherence between available epidemiological data and dietary intervention data underscores the requirements for further studies to unravel other non-lycopene components in tomato and their roles in CVD risk reduction. Tomatoes contain several known and unknown compounds that might affect platelet function, lipid metabolism, blood pressure and endothelial function, important determinants of CVD [5, 39]. Lycopene or other antioxidants act through inhibition of LDL oxidation alone and so may only partly contribute to the CVD risk reduction. Recent studies showed that tomato also contains gamma-aminobutyric acid, 13-oxo-9,11-octadecadienoic acid and esculeoside A, which may provide heart and psychological health benefits [40]. 13-oxo-ODA is, however, found only in tomato juice [41]. Esculeoside A reduces plasma lipids and therefore may ameliorate atherosclerotic lesions in ApoE-deficient mice [42]. This indicates that other unidentified compounds (non-lycopene compounds) may also contribute to the cardio-protective effects of tomatoes as observed in epidemiological and interventional studies.

## Role of platelet hyperactivity in the development of CVD

Platelet activity is thought to play a major role in the development as well as the stability of atherosclerotic plaques. Abnormal activation of blood platelets might represent a contributory risk factor for accelerated vascular disease, which occurs in hypertension, because it plays an important role in the development and progression of atherosclerosis [43]. In support of the pathophysiological role of platelets, platelet inhibitory drugs such as aspirin have been observed to reduce the incidence of myocardial infraction, stroke and death from CVD in secondary prevention trials [11, 43]. The pathophysiological state of platelets (hyperactive) is the underlying risk of problems in diabetes, smoking, obesity and sedentary lifestyle and other conditions (Table 1). Although the mechanism of the increase in platelet reactivity is uncertain in these conditions, it could be caused by sensitization of platelets to aggregation by elevated levels of agonists in vivo [44], or it could be due to the redistribution of young, more reactive platelets that are concentrated in the spleen [45] and are released into the systemic circulation. Platelets are activated by a large number of agonists that are released in the circulation during some pathologic conditions (e.g. hypertension and diabetes mellitus) [46]. Several lines of evidence point to an important role for platelets in the pathogenesis of sudden death, acute myocardial infarction and unstable angina [47]. Other evidence supporting a causal role of local platelet activation in the pathogenesis of acute coronary events comes from studies demonstrating that aspirin, an inhibitor of platelet thromboxane A_2_ (TxA_2_) synthesis, reduces the incidence of acute myocardial infarction and of cardiac death in patients with unstable angina. Recently, aspirin’s antiplatelet limitations have progressively underscored the critical need for improved platelet aggregation inhibitor therapy which is not only effective, but also safe and well tolerated [11, 48, 49]. This concept has stimulated research into prevention of platelet hyperactivity by several means including dietary supplementation. Development of the atheroma is a gradual process, understood to be influenced by well-established traditional risk factors, including (but not limited to) hypertension, cigarette smoking, diabetes mellitus, dyslipidemia and a family history of premature atherosclerotic disease. Considerable investigation into the pathophysiological effects induced by these factors has elucidated their contributions to the prothrombotic milieu within the atherosclerotic coronary artery preceding an atherothrombotic event. Activated platelets release different growth factors (e.g. PDGF and VEGF) that participate in the development of atherosclerosis by promoting VSMC proliferation [50]. Stimulated platelets release VEGF [51], and elevated VEGF levels have been found in patients with atherosclerotic risk factors such as hypertension [52]. Tests of platelet function have been used to investigate a possible role of platelet hyper-reactivity in the pathogenesis of vascular disorders and their complications. Platelet activation and aggregation are also involved in the development of hypertension in different ways. Activated platelets release different mediators, such as 5-hydroxytryptamine (5-HT or serotonin), ADP, ATP and lysophosphatidic acid [50]. A number of these agents enhance the intracellular Ca^2+^ concentration ([Ca^2+^]_i_) in vascular smooth muscle cells (VSMC), which promotes vasoconstriction and increases catecholamines response. Furthermore, the number of platelet α-adrenergic receptors increases in hypertensive persons [53], which may promote catecholamines responses. Catecholamines, β-adrenoceptor agonist isoprenaline and angiotensin II (Ang II) increase [Ca^2+^]_i_ and promote contraction of VSMC, platelet activation and aggregation [53] which may participate in the genesis and maintenance of hypertension. Moreover, it has been shown that Ang II increases [Ca^2+^]_i_ and pH in platelets from hypertensive patients, which may be associated with enhanced platelet aggregation [54]. In hypertension, platelets showed spontaneous aggregation and increased sensitivity to agonists [55, 56]. Furthermore, platelets release more β-thromboglobulin and P-selectin and have higher intracellular Ca^2+^ levels [57, 58]. Hypertension is associated with oxidative stress [57]. Thus, in hypertensive patients, platelets produce more reactive oxygen species which enhance platelet activity by reducing the bioavailability of nitric oxide (NO) and enhancing [Ca^2+^]_i_ among other cellular effects. Platelets are directly influenced by specific adipokines and therefore have the potential to serve as an essential mediator of the cardiovascular consequences of obesity. Consistent with this, obesity has been associated with increases in platelet aggregation, elevations in surface expression of markers of platelet activation such as P-selectin and heightened platelet microparticle formation. More importantly, reduction in adipose mass leads to normalization of markers of enhanced platelet activation. However, a causal role for platelet hyperactivation in obesity-related cardiovascular disorders remains to be established. Several characteristics and proven biological activities of platelets make them an appealing candidate for triggering and maintaining the inflammatory response of obesity. Activated, but not resting platelets are able to alter the chemotactic properties of endothelial cells by inducing the secretion of monocyte chemoattractant protein. Similarly, transforming growth factor-β is released from activated platelet α-granules and has been shown to augment the release of type-1 plasminogen activator inhibitor from adipose tissue. Importantly, a recent report indicates that the recruitment of inflammatory cells in adipose tissue is facilitated by platelet adhesion along activated endothelium. Based on these observations, it seems that platelet activation, secondary to obesity, plays a causal role in triggering and maintaining the pro-inflammatory and pro-thrombotic state of obesity, creating a feedback loop involving adipose tissue, activated platelets and vascular endothelium that culminates in an environment favourable for atherothrombotic vascular events [59, 60]. Therefore platelet activation contributes to the inflammatory and thrombotic consequences of obesity. All the data indicate the importance of taming platelets in order to avoid CVD.

**Table 1:** Hyperactivity of platelets related to disease

Conditions lead to hyperactive platelets
Diabetes mellitus
Insulin resistance
Obesity
Ageing
Over nutrition, bad diets
Sedentary lifestyles
Oxidative stress, inflammation and hyperlipidemia
Drugs, contraceptives
Cancers
Hypertension

Platelets become hyperactive or produce circulating micro-aggregates in the clinically defined conditions shown

## In vitro studies with water-soluble tomato extract on human blood platelet aggregation

The antiaggregatory effects of different aqueous fruit extracts on human platelets in vitro have been published previously [13]. The maximum inhibitory effect (70–75 %) was found to be with tomato and kiwi fruit extracts, whereas apple and pear had very little activity (2–5 %). Grapefruit, melon and strawberry had intermediate activities on platelet aggregation (33–44 %). The antiplatelet potential of the fruits tested appeared to have no relationship with their antioxidant activity [13]. These antiplatelet compounds in tomato had a molecular mass less than 1000 Da and were highly water soluble and stable to boiling. The compounds of interest were concentrated into an aqueous extract produced by homogenizing fresh tomatoes, removing particulate matter and delipidating. The delipidated aqueous extract was then further fractioned by gel filtration using a Biogel P2 column [13]. Adenosine, a known antiplatelet factor, was identified in one fraction, but its removal from the whole extract did not substantially decrease the antiplatelet activity, indicating the presence of additional, different antiplatelet agents. Further work showed that the aqueous tomato extract consisted largely of soluble sugars (85–90 % of dry matter), which showed no in vitro antiplatelet activity [15]. The non-sugar material that was isolated (tomato total active fraction, tAF) accounted for 4 % of the aqueous tomato extract dry matter and showed strong inhibition of platelet aggregation in vitro. Isolation of many individual components from tAF followed, and it was found that most fell into one of three categories—nucleosides, simple phenolic derivatives and flavonoid derivatives. All showed antiplatelet activities consistent with their compound categories.

**Fig. 2:**
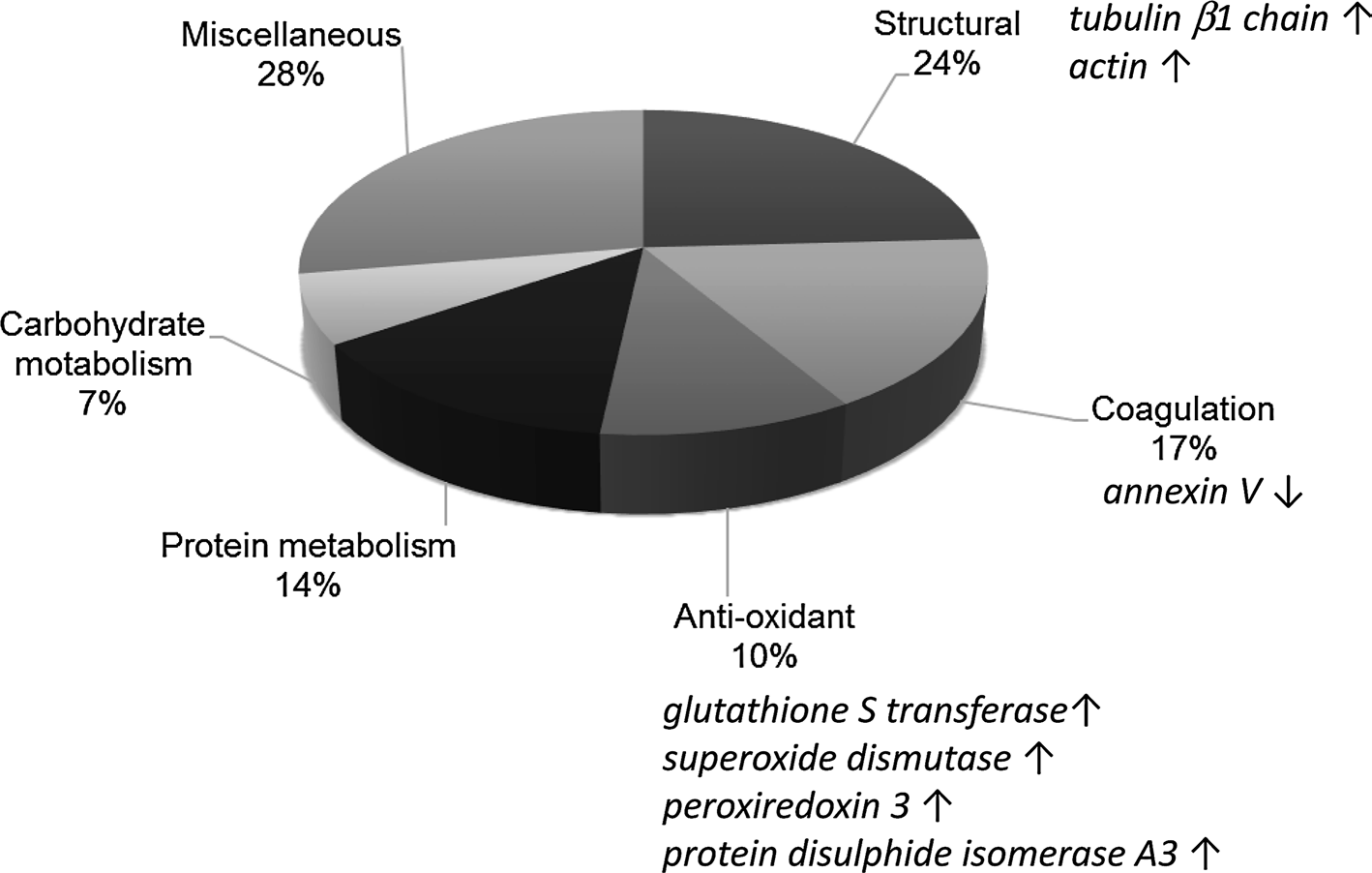
Effects of Fruitflow^®^ on the platelet proteome. Classes of protein showing statistically significant changes after treatment with Fruitflow^®^ at a concentration of 43 mg L^−1^ (maximum theoretical circulating concentration after ingestion of a single 3 g/150 mg dose equivalent to 65 mg tAF or approximately three bowls of tinned tomato soup)

Proteomic experiments carried out to examine effects of tAF on platelet signalling pathways showed that tAF components altered a range of platelet functions including those regulating platelet structure, coagulation and redox status (Fig. 2). One of the most strongly affected proteins was protein disulphide isomerase (PDI), an oxidoreductase which catalyses the formation and the isomerization of disulphide bonds. In platelets, blocking PDI with inhibitory antibodies inhibits a number of platelet activation pathways, including aggregation, secretion and fibrinogen binding [61, 62]. Other investigators [63] have reported similar functional effects after blockage of cell surface thiol isomerases. Glycosides related to quercetin, of which several are present in tAF [O’Kennedy N. Fruit extracts. International Patent WO 2010/049707, May 06, 2010], have been shown to interact with PDI in this way [64, 65]. Interaction of polyphenols with PDI suggested a possible mechanism by which tomato extract components could inhibit different pathways of platelet aggregation.

The functional effects of tAF components were therefore examined in a series of experiments. tAF and its sub-fractions F1, F2 and F3, prepared by semi-preparative reversed-phase HPLC as described by O’Kennedy et al 2006 [66], were observed to prevent activation of integrin αIIbß3 (ie, GPIIb/IIIa). Inhibition of the GPIIb/IIIa activation step—which is common to multiple aggregation pathways—could underlie the wide-ranging effects of tAF [15]. This is consistent with the observation that basal platelet cyclic AMP concentrations (controlled by phospholipase C enzyme family-mediated cascade reactions) are unaltered by tomato extract active components in vitro. In addition, tAF reduced the expression of P-selectin (CD62P) on the platelet surface in response to ADP-induced platelet activation in whole blood [15, 66]. In resting platelets, P-selectin is localized in the membranes of platelet α-granules. On platelet activation, it is redistributed to the platelet surface, where it initiates adhesion to leucocytes. Under conditions of blood flow and shear stress, this glycoprotein promotes platelet cohesion and stabilizes newly formed aggregates. Thus, tAF components can potentially affect the size and longevity of platelet aggregates. tAF components were also found to affect the binding of tissue factor (TF) to activated platelets, at least in part due to effects on P-selectin [O’Kennedy N and Song H-J. Therapeutic uses of tomato extracts. International Patent WO 2007/141495, December 13, 2007].

**Fig. 3:**
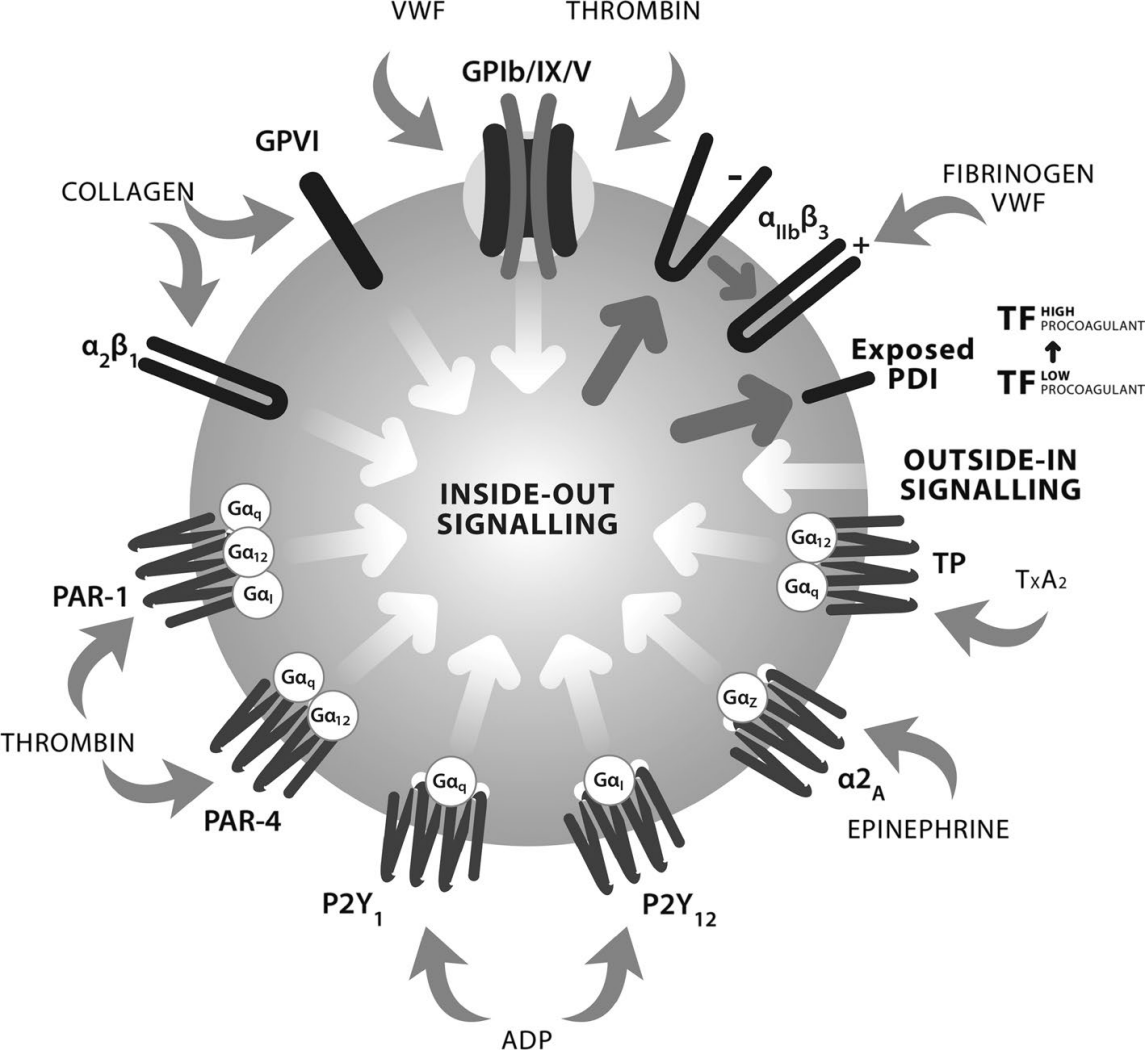
Platelet activation via multiple pathways and sites of action of Fruitflow^®^ ingredients. Key agonists, their receptors and triggering signalling pathways involved in platelet activation and subsequent aggregation. *VWF* von Willebrand factor, *TXA2* thromboxane-A2, *TP* thromboxane receptor, *ADP* adenosine diphosphate, *PAR* proteinase-activated receptor, *PDI* protein disulphide isomerase, *TF* tissue factor. The activation of platelets is accompanied by a conformational change in integrin aIIbb3 (glycoprotein (GP) IIb–IIIa), exposing a binding site for fibrinogen and triggering the release of aggregating agents such as TXA2 and ADP. Adenosine nucleotides signal through P2 purinergic receptors (P2Y) on the platelet membrane. Activation of these receptors initiates a complex signalling cascade that ultimately results in platelet activation, aggregation and thrombus formation. Thrombin acts via cell surface protease-activated receptors (PARs). Both stimulate PLC giving rise to PIP2 hydrolysis and consequent activation of PI3K. Collagen acts both through receptors (GPIV) and on integrin a2b1, promoting adhesion and triggering inside-out signalling. Inside-out signalling alters platelet skeletal characteristics to allow spreading and microparticle release, leading to increase in pro-coagulant potential. Inside-out signalling also exposes PDI on the platelet surface, which can alter the pro-coagulant potential of TF in situ, or alternatively transfer to endothelial cells. Fruitflow components have been shown to affect ADP, collagen, thrombin and TXA2-mediated signalling, to affect integrin activation and subsequent fibrinogen binding, and to down-regulate platelet PDI

In summary, these results demonstrating the actions of tAF on different platelet functions were all consistent with potential effects mediated partly through polyphenols and PDI and partly through nucleosides elevating cAMP and cGMP levels in platelets [13, 67]. Effects on TF binding suggested that tAF components could have a larger effect on some aspects of the coagulatory response, such as thrombin generation, than previously imagined. Figure 3 summarizes the actions of different ingredients of Fruitflow^®^ on platelet activation pathway.

## Development of Fruitflow^®^: Compositional and structural aspects

To develop prototype plant-derived antiplatelet extracts in the laboratory requires understanding of the extract components and the structure–function relationships affecting their mechanism of action and potency. These details were largely established for aqueous tomato extracts during an exploratory research phase as discussed earlier. However, to move from the laboratory prototype to a standardized food ingredient required a change in focus to extract reproducibility, cost analysis and development of a rigorous quality assurance system capable of standardizing bioactivity and controlling raw material variation, processing-induced effects and multiple sites of manufacture. Focusing on these points developed the small-scale laboratory-grade aqueous tomato extract into a commercial food grade tomato extract, which was given the trade name Fruitflow^®^.

Today, Fruitflow^®^ is made in two ingredient formats. The raw material for both formats is high grade, minimally processed tomato commodity products. Fruitflow^®^ 1 is a syrup of which more than 50 % w/w comprises tomato-derived carbohydrates, and ~3 % w/w comprises known bioactive compounds with measured antiplatelet activity. This ingredient format is especially suitable for use in drinks and foods with high water content. Fruitflow^®^ 2 is a low-carbohydrate powder, of which more than 55 % w/w comprises bioactive compounds, dried to produce a tablet-grade powder. Fruitflow^®^ 2 can be compressed into tablets and has flow properties which render it suitable for capsule formation or for use in dry-blend food processes. Both ingredient formats are lycopene- and fat-free, low in inorganic salts and low in organic acids. The potency of Fruitflow^®^ 1, in terms of bioactive compound content/g, is lower than that of Fruitflow^®^ 2, as a consequence of their different sugar content: relative potencies of the two ingredients when compared w/w are in an approximate ratio of 1:20. Thus, 3 g Fruitflow^®^ 1 gives an equivalent dose of bioactives (approximately 65 mg) to 150 mg Fruitflow^®^ 2. Of this quantity, 6–10 % (up to 9 mg) are known nucleoside derivatives (F1), 13–15 % (up to 10 mg) are known phenolic conjugates (F2), and 8–10 % (up to 7 mg) are known flavonoid derivatives (F3), including a minimum of 2.4 mg quercetin derivatives per dose. This amount of bioactive compounds, which comprises a single daily dose, is equivalent to that found in about 3 average servings of tinned tomato soup.

The development of a quality assurance system which allows such standardization began with the implementation of an analytical HPLC system simple enough to run on the factory floor, but precise enough to measure selected important antiplatelet compounds through each process step. The individual bioactivity profiles of compounds occurring in Fruitflow^®^ were examined, and three representative components, one from each of the three broad fractions F1, F2 and F3 (as discussed earlier, these fractions were originally defined by reverse-phase HPLC fractionation, described in [15]), were selected. These three compounds were adenosine, which represents a group of nucleosides/nucleotides found in F1, chlorogenic acid, which represents a group of phenolic derivatives found in F2, and rutin, which represents a group of flavonoid derivatives found in F3. The representative compounds were monitored throughout the developing process, from raw material to final product; losses in any one of them would reflect losses of the entire class of compounds each represented and could be quantified and assessed. In addition, total tAF content was quantified throughout. The data collected were mapped onto bioactivity (antiplatelet activity) and the validity of the measurements as a method of monitoring overall bioactivity established. The use of this bioactivity-led analysis then facilitated selection of suitable raw materials, control of raw material variability and investigation of the effects of thermal processing on bioactivity of extracts produced.

The presence within tomatoes of such a range of structurally different water-soluble secondary metabolites reflects both the complex sensory profile of the tomato and its response to stresses. Many of the bioactive compounds are produced in the tomato fruit as part of the plant defences against insect or bacterial challenges. However, as tomatoes are grown under a wide range of conditions worldwide, a similarly wide variability in secondary metabolite composition might be expected. Table 2 shows the content of antiplatelet compounds (tAF) in tomato juice and the overall antiplatelet activities of the juice, from a range of different cultivars, geographical sources and degrees of ripeness. While tomatoes from different geographical locations and of different cultivars did show differences in the quantity of antiplatelet compounds extracted, the degree of ripeness of the tomatoes appears to be the most important factor in determining bioactivity. Thus, ripeness of tomatoes at the time of harvest became a key quality assurance factor in the development of the Fruitflow^®^ food ingredients.

**Table 2:** Influence of tomato cultivar and ripeness on yield and antiplatelet activity of tAF

Source	Cultivar	Ripeness	tAF isolated (mg/kg tomato fresh wt)	tAF IC50 against ADP agonist (mg/mL PRP)
UK	Fresh market cultivar (greenhouse grown)	Green	212 ± 50	10.8
		Fully ripe	533 ± 35	<1.0
	Processing cultivar E6203 (greenhouse grown)	Green	251 ± 64	15.6
		Fully ripe	600 ± 77	<1.0
Spain	Processing cultivar	Green	377 ± 35	13.9
		Fully ripe	745 ± 43	<0.5
	Processing cultivar	Green	352 ± 65	15.9
		Fully ripe	698 ± 23	<0.5
Chile	Processing cultivar (tunnel grown)	Green	303 ± 54	12.7
		Fully ripe	712 ± 33	<0.5
	Processing cultivar (tunnel grown)	Green	364 ± 12	10.3
		Fully ripe	768 ± 42	<0.5

Cultivars were largely selected from typical processing cultivars for the region, but one fresh market cultivar was included. Quantities are given as mean values ± SEM

The type of processing undergone by fresh tomato fruit determines the extent to which the original antiplatelet activity of the fresh fruit can be retained. High-temperature processing and/or long holding times at moderate–high temperatures are detrimental to the antiplatelet activities of final products. Table 3 shows the tAF quantified in a range of processed tomato foods. Many of the products tested displayed a very low tAF content and a correspondingly low bioactivity. It became evident from this work that control of temperature and time at temperature during processing was a key to retaining bioactivity in any process attempting to make a concentrated extract from tomato juice. On this basis, after a survey of available materials, cold break (low heat treatment), minimally processed tomato products were chosen as suitable starting materials for a high antiplatelet activity tomato extract. Once the starting material and its quality controls had been established, the Fruitflow^®^ 1 and 2 extraction processes were designed and optimized, with reference to content of tAF and the three representative bioactives, in a way that directly optimized bioactivity. The key features of both processes were control of time spent at high temperatures and the balance necessary to achieve the desired temperature profile while avoiding microbiological and waste issues. Fruitflow^®^ 1 is currently produced from cold break minimally processed tomato products (pastes, passatas, juices) in a 5-step process, which utilizes physical separation methods (centrifugation, filtration) at low temperatures to remove pulp and unwanted insoluble solids from the starting material, leading to a clear yellow juice. This is then concentrated by low-temperature evaporation and standardized for pH.

**Table 3:** Quantities of tAF components (shown separated into fractions F1, F2 and F3) isolated from a range of processed tomato products

Product	F1		F2		F3	
	Mean	Range	Mean	Range	Mean	Range
Fresh tomato extract	8095.6	n/a	410.3	n/a	1802.3	n/a
Commercial tomato paste*	6014.2	4211.8–7207.1	263.0	22.9–433.6	1176.3	945.5–1404.9
Commercial tomato soup*	3813.3	1089.1–5685.1	318.2	24.1–400.2	767.4	241.5–1586.6
Commercial tomato juice*	5661.5	5649.1–5692.0	84.0	54.5–99.3	1146.3	896.4–1377.9
Commercial tomato sauce*	4680.5	3599.8–5156.2	139.8	92.4–93.7	1155.8	823.8–1787.2
Fruitflow^®^	7874.6	n/a	389.2	n/a	2141.3	n/a

The various tomato products were prepared by dilution with water, centrifugation and filtration to remove all insoluble materials, and freeze drying to yield dried soluble solids. The soluble solids were then redissolved in water and standardized to a set concentration before extraction of tAF using solid-phase extraction and separation of F1, F2 and F3 by semi-preparative reversed-phase HPLC. Values shown are expressed in μg g^−1^ product soluble solids and represent the mean quantities of F1, F2 and F3 isolated within product categories, together with the observed range for each category

* *n* = 10 products

Fruitflow^®^ 2 is a low-sugar derivative of Fruitflow^®^ 1, deriving from the same raw material and using the same initial clarification processing steps. After removal of insoluble material, complex and simple sugars are extracted from the clarified tomato juice using an adsorption column process, in which the sugars which are the major constituents of tomato juice pass through a resin column without retention, while the remainder of the juice components are adsorbed from the juice and retained on the column. These retained components of interest—which can represent up to 2 % of the fresh weight of the tomato-derived starting material—are then recovered from the column, and the concentrate is dried to powder and standardized for moisture content. The resulting Fruitflow^®^ 2 powder contains almost no sugars (glucose, fructose, pectin, starch or derivatives). The bioactive components are present in concentrations between 28–32 times higher than in Fruitflow^®^ 1, due to the removal of the sugar and water matrix of the syrup ingredients. However, as the resin column used successfully retains the remainder of the tomato juice constituents, the relative occurrence of these components in Fruitflow^®^ 2 remains similar to that in Fruitflow^®^ 1 (i.e., the process does not significantly alter the relative proportions of the bioactive components).

An industrial specification for each ingredient ensures that the final product of each process is an extract whose antiplatelet activity against ADP, collagen, AA and TRAP in vitro is above a minimum level (Table 4) and as close to fresh tomato juice as possible. This specification is achieved by the monitoring of tAF and the three compounds adenosine, chlorogenic acid and rutin, representatives of F1, F2 and F3 in the fresh tomato. The specification allows a standardized usage of the ingredients, so that Fruitflow^®^ 1 and 2 may be used interchangeably depending on the application.

**Table 4:** Antiplatelet activity of the standardized Fruitflow^®^ 1 and Fruitflow^®^ 2 ingredients, expressed in terms of their IC50 values (mg Fruitflow/mL platelet-rich plasma) with regard to the major platelet agonists ADP, collagen, thrombin (thrombin receptor-activated peptide, TRAP, used) and arachidonic acid

	IC50 ADP	IC50 Collagen	IC50 TRAP	IC50 AA
Antiplatelet activity of the standardized Fruitflow^®^ 1 and Fruitflow^®^ 2 ingredients, expressed as IC50 values				
Fruitflow^®^ 1	<1.0	<1.0	<2.7	<3.6
Fruitflow^®^ 2	<0.05	<0.05	<0.5	<0.7

The IC50 in this context represents the amount of Fruitflow^®^, in mg dry matter, needed to inhibit platelet aggregation in 1 mL PRP by 50 %, compared to control (saline) treatment. All values given are averages of triplicate measurements taken in PRP harvested from 10 different platelet donors

The Fruitflow^®^ 1 and 2 ingredients are now incorporated into a range of different food products, ranging from drinks to tablets. Both ingredients are stable in a wide variety of matrices, with appropriate shelf life in all, tested to industry standard ICH levels.

## Human trials undertaken with Fruitflow^®^ 1 and Fruitflow^®^ 2

### Early studies

**Fig. 4:**
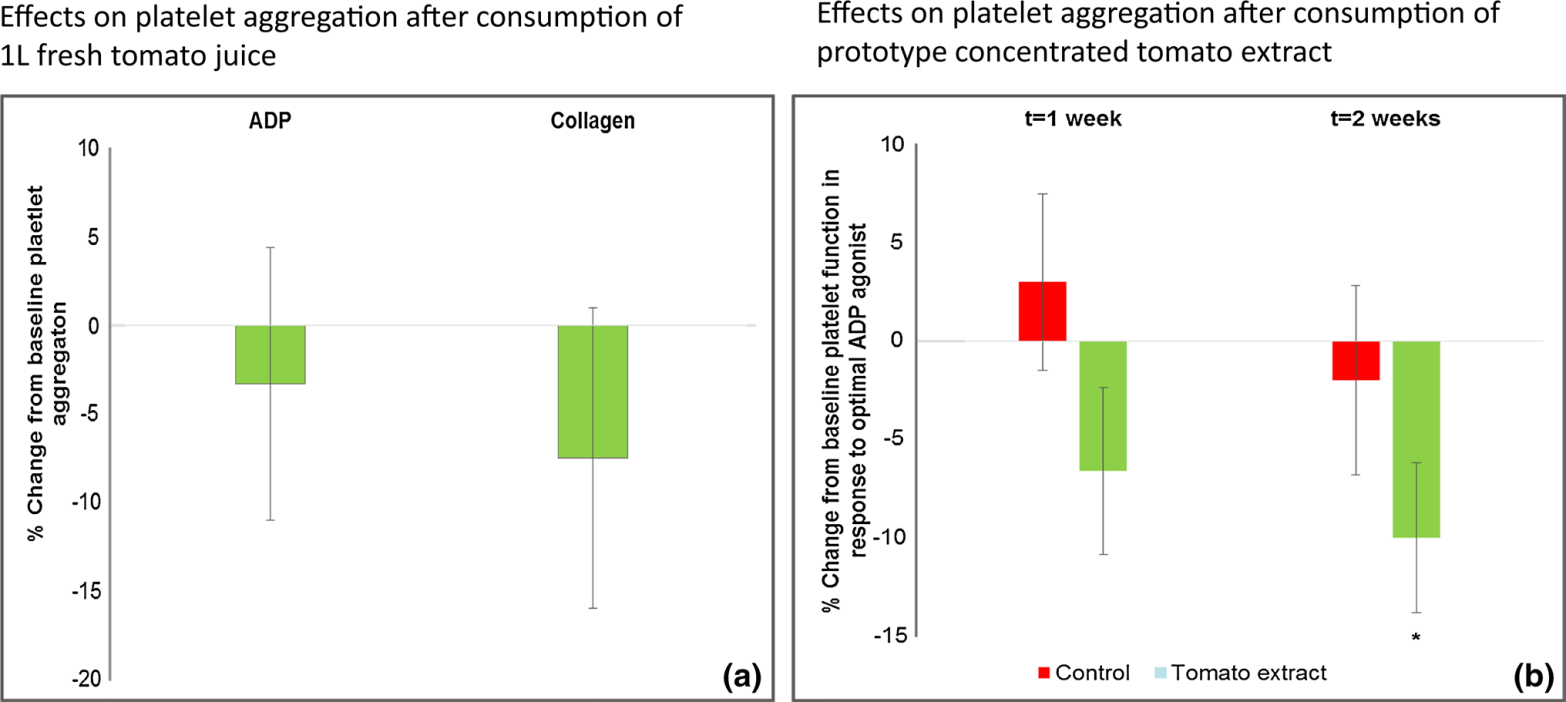
Summary of results from early-stage studies using tomato extract prototypes. **a**  % Change from baseline aggregation observed after ingestion of 1L freshly produced, filtered tomato juice, by healthy subjects (*n* = 9). This was a pilot study and was not placebo-controlled. Changes from baseline aggregation in response to ADP and collagen were not significant. **b**  % Change from baseline aggregation observed after consumption of either a prototype concentrated tomato extract or control extract for a period of two weeks. The study involved healthy subjects (*n* = 14). Changes from baseline aggregation in response to optimal ADP agonist (concentration used titred to give optimal platelet response in each sample) were significantly different from control at* t* = 2 weeks

The first study which investigated the antiplatelet effects of a water-soluble tomato extract used freshly prepared tomato juice. As this juice was low in tAF concentration, a volume of 1L was given to ensure sufficient ingestion of tAF components. No significant effects could be observed in the small study group (*n* = 9, Fig. 4a). However, a second study using a crudely prepared prototype tomato extract concentrate, in which the subjects consumed approximately the same dose of bioactive compounds as in the 1L study, but in a much smaller volume (~10—12 mL), did show an observable effect on platelet function at a variety of times after supplementation (Fig. 4b). It was concluded that tomato juice would be an inappropriate vehicle for its antiplatelet compounds when attempting to achieve a systemic acute antiplatelet response via oral ingestion, and work began to refine the crude prototype already tested.

## Main body of studies undertaken with standardized Fruitflow^®^ 1 and Fruitflow^®^ 2

After development of the Fruitflow^®^ ingredients, and standardization of testing conditions, a unified set of studies was carried out to establish the efficacy of these novel ingredients *ex vivo*. A summary of the studies in which the standardized ingredients Fruitflow^®^ 1 and Fruitflow^®^ 2 have been used is given in Table 5. This table also allows comparison of the major outcomes of each of these studies, in terms of the extent of the antiplatelet effects measured in comparison with the study baseline and to the study control. Studies which established the onset time of an acute antiplatelet effect after oral ingestion of a dose of Fruitflow^®^ 1 have been published elsewhere [15]. These studies showed that in all subjects, an acute lowering of platelet aggregability to ADP and collagen was observed at a time three hours after consuming Fruitflow^®^ (Table 5, #1). The range of onset times was from one and a half hours to three hours after consumption. In contrast, the normal diurnal increases in platelet aggregability were illustrated in subjects consuming the control supplement over the time course measured. The persistence of this acute effect varied between individuals, but in all cases platelet aggregability returned to baseline 18 h after consumption of a single dose of Fruitflow^®^ [68].

**Table 5:** Summary of human studies undertaken during the development of the Fruitflow^®^ ingredient family

Study	Description	Main purpose	N/gender/study population	Duration /intervention	Outcome measures	Major results for intervention expressed as % change from baseline measurements at* t* = 3 h after supplementation, unless otherwise stated		
						Aggregation	Clotting times	Other
1. O’Kennedy et al. 2006a	R, SB, PC crossover	Acute effect, onset and persistence	27/M F healthy	Single dose Control (treacle) or FF1 equivalent to three daily doses, 195 mg tAF, in 50 mL or 200 mL OJ	Platelet aggregation Clotting times	*C 50* *mL:* ADP: 6.8 (1.5) *C 200* *mL:* ADP: 2.9 (3.4) *FF 195* *mg tAF 50* *mL:* ADP: −23.1 (9.8)*P*<0.001,* *FF 195* *mg tAF 200* *mL:* ADP: −17.8 (5.1)*P*<0.001,*	*C 50* *mL*: PT: 0.3 (2.7) TCT: 1.9 (1.8) *C 200* *mL:* PT: 0.3 (2.7) TCT: 1.9 (1.8) *FF 195* *mg tAF 50* *mL:* PT: 0.8 (1.8) TCT: −2.9 (1.2) *FF 195* *mg tAF200* *mL:* PT: 0.8 (1.8) TCT: −2.9 (1.2)	
2. O’Kennedy et al. 2006b	R, DB, PC crossover	Acute effect, size of effect, dose–response	93/M F healthy	Single dose Control (treacle) in 200 mL juice or FF1 equivalent to one daily dose, 65 mg tAF in 200 mL juice or FF1 equivalent to three daily doses, 195 mg tAF, in 200 mL juice	Platelet aggregation Clotting times	*C:* ADP: −2.6 (2.9) Coll: −7.3 (2.4) *FF 65* *mg tAF:* ADP: −12.7 (3.1)^*P*<0.01,*^ Coll: −14.5 (2.5)^*P* = 0.003,*^ *FF 195* *mg tAF:* ADP: −21.3 (2.9)^*P*<0.001,*^ Coll: −18.0 (2.8)^*P*=0.003,*^	*C:* PT: 0.8 (0.7) TCT: 1.8 (1.2) *FF 65* *mg tAF:* PT: 3.5 (2.4) TCT: 0.2 (1.1) *FF 195* *mg tAF:* PT: 0.1 (0.7) TCT: 1.2 (1.3)	
3. O’Kennedy et al (unpublished)	R, DB, PC crossover	Chronic effect	22/M F healthy	28d daily supplementation with samples every 14 days Control (treacle) in 200 mL juice or FF1 equivalent to one daily dose, 65 mg tAF, in 200 mL juice	Platelet aggregation Clotting times	*C 14d:* ADP: −1.5 (5.5) *FF 65* *mg tAF 14d:* ADP: −19.0 (4.1)^*P*=0.0004,*^ *FF 65* *mg tAF 28 d:* ADP: −28.2 (4.1)^*P*=0.0001,*^	*C 14d:* PT: 0.0 (1.5) TCT: 2.3 (3.0) *FF 65* *mg tAF 14d:* PT: −3.3 (1.5) TCT: 3.1 (3.0) *FF 65* *mg tAF 28 d:* PT: −1.4 (1.5) TCT: −2.7 (3.0)	
4. O’Kennedy et al (unpublished)	R, DB, PC crossover	Acute effect, different formats of FF (liquid and powder forms)	45/M F healthy	Single dose Control (treacle and cellulose powder) or FF1, FF2a or FF2b equivalent to one daily dose, 65 mg tAF, given as capsules	Platelet aggregation Clotting times TxA2 generation in response to collagen	*C:* ADP: −8.7 (3.9) Coll: −0.9 (2.9) *FF1 65* *mg tAF:* ADP: −17.2 (3.7)^*P*<0.001,*^ Coll: −15.6 (2.6)^*P*<0.001,*^ *FF2a 65* *mg tAF:* ADP: −15.5 (3.8)^*P*<0.001,*^ Coll: −14.4 (2.8*)* ^*P*<0.001,*^ *FF2b 65* *mg tAF:* ADP: −13.5 (3.8)^*P*<0.001,*^ Coll: −11.6 (2.7*)* ^*P*<0.001,*^	*C:* PT: 0.4 (0.7) TCT: 0.6 (1.3) *FF1 65* *mg tAF:* PT: 0.3 (0.7) TCT: 0.5 (1.3) *FF2a 65* *mg tAF:* PT: 0.6 (0.7) TCT:0.4 (1.3) *FF2b 65* *mg tAF:* PT: 1.3 (0.8) TCT: 0.1 (1.3)	*C:* TxB2: 6.8 (8.3) *FF1 65* *mg tAF:* TxB2: −25.1 (8.3)^*P*=0.0008,*^ *FF2a 65* *mg tAF:* TxB2: −33.5 (8.3)^*P*=0.0003,*^ *FF2b 65* *mg tAF:* TxB2: −36.8 (8.3)^*P*<0.0001,*^
5. O’Kennedy et al (unpublished)	R, positive C crossover	Over-consumption safety study	20/MF healthy	Single dose and 5d daily dose FF1 equivalent to one daily dose, 65 mg tAF, given in 250 mL juice, or FF1 equivalent to four daily doses, 260 mg tAF, given in 1L juice either once or once per day for 5 days.	Platelet aggregation Clotting times	*FF 65* *mg tAF in 250* *mL:* ADP: −13.0 (2.8)^*P*=0.0001^ TRAP: −17.9 (4.0)^*P*<0.0001^ *FF 260* *mg tAF in 1L:* ADP: −3.1 (2.8)^*^ TRAP: 1.2 (3.6)^*^ *FF 260* *mg tAF in 1L, 5d:* ADP: −3.5 (2.7)* TRAP: −0.6 (4.1)*	*FF 65* *mg tAF in 250* *mL:* PT: −0.3 (0.5) TCT: 0.7 (0.6) *FF 260* *mg tAF in 1L:* PT: −0.5 (0.4) TCT: −0.9 (0.6) *FF 260* *mg tAF in 1L, 5d:* PT: −1.0 (0.4) TCT: −0.5 (0.5)	
6. O’Kennedy et al (unpublished)	R, positive C crossover	Matrix effects	9/MF healthy	Single dose FF2 equivalent to one daily dose, 65 mg tAF, in juice (positive control) or FF2 equivalent to one daily dose, 65 mg tAF, in yoghurt or FF2 equivalent to four daily doses, 260 mg tAF, in yoghurt	Platelet aggregation Clotting times	*FF2 65* *mg tAF (juice):* ADP: −18.4 (8.2)^*P*<0.05^ Coll: −18.0 (6.3)^*P*<0.016^ *FF2 65* *mg tAF (yoghurt):* ADP: −13.7 (5.6)^*P*=0.033^ Coll: −9.4 (5.3) *FF 260* *mg tAF (yoghurt):* ADP: −25.7 (7.6)^*P*=0.006^ Coll: −12.4 (5.3)^*P*=0.010^	*FF2 65* *mg tAF (juice):* PT:0.1 (0.5) TCT: −0.3 (1.6) *FF2 65* *mg tAF (yoghurt):* PT: −0.1 (0.5) TCT: 0.7 (1.6) *FF 260* *mg tAF (yoghurt):* PT: 0.2 (0.5) TCT: −0.3 (1.6)	
7. O’Kennedy et al (submitted for review)	R, DB, PC crossover	Comparison of a single dose of FF to a single dose of 75 mg ASA, and to 7d of 75 mg ASA	47/MF healthy	Single dose and 7d supplement Control (treacle or cellulose powder) either as a single dose or once daily for seven days or FF1 equivalent to one daily dose, 65 mg tAF, given as a capsule or 75 mg ASA given as a capsule or 75 mg ASA given as a capsule once daily for 7 days	Platelet aggregation Clotting times TxA2 generation in response to collagen/AA PFA − 100 closure time	*C:* ADP:0.8 (4.8) Coll: −3.1 (3.9) AA: −0.6 (5.6) *FF 65* *mg tAF:* ADP: −26.3 (5.3)^*P*=0.005, *^ Coll: −9.7 (4.2)^*P*=0.018, *^ AA: −15.8 (6.0)^*P*<0.001^ *ASA 75* *mg single dose:* ADP: −26.7 (5.9)^*P*<0.001, *^ Coll: −23.5 (4.6)^*P*<0.001, *^ AA: −56.5 (6.4)^*P*<0.001, *^ *ASA 75* *mg 7d:* ADP: −26.3 (5.3*)* ^*P*<0.001, *^ Coll: −49.8 (4.2)^*P*<0.001, *^ ASA: −88.5 (6.0)^*P*<0.001, *^	*C:* PT: −0.8 (1.7) TCT: −1.4 (1.1) aPTT: 0.0 (1.2) *FF 65* *mg tAF:* PT: 1.1 (1.1) TCT: 1.1 (1.1) aPTT: 1.7 (1.3) *ASA 75* *mg single dose:* PT: −3.5 (1.9) TCT: −1.3 (1.2) aPTT: 1.4 (1.4) *ASA 75* *mg 7d:* PT: −0.8 (1.8) TCT: −0.5 (1.2) aPTT: 1.2 (1.3)	*C:* TxB2 (coll): −2.2 (5.8) TxB2 (AA): −1.74 (6.5) PFA-100: 5.9 (8.4) *FF 65* *mg tAF:* TxB2 (coll): −34.1 (6.3)^*P*<0.001, *^ TxB2 (AA): −39.8 (6.8)^*P*<0.001, *^ PFA -100: 47.6 (8.4)^*P*<0.001, *^ *ASA 75* *mg single dose:* TxB2 (coll): −69.3 (6.8)^*P*<0.001, *^ TxB2 (AA): −62.8 (7.2)^*P*<0.001, *^ PFA -100: 56.7 (8.4)^*P*<0.001, *^ *ASA 75* *mg 7d:* TxB2 (coll): −91.4 (6.3)^*P*<0.001, *^ TxB2 (AA): −99.0 (6.9)^*P*<0.001, *^ PFA-100: 127.8 (10.4)^*P*<0.001, *^

All studies listed were undertaken with a standardized Fruitflow^®^ ingredient so that a single daily dose approximated 65 mg tAF components in all cases. This can be considered approximately equal to the intake consumed with three bowls of tinned tomato soup. Studies are described in terms of design (randomized (R), single blinded (SB), double blinded (DB), placebo controlled (PC), positive control (positive C), crossover or parallel group), main purpose, study population and number of participants (N), and duration of intervention. The major outcome measures are described, and results (mean values with SEM in brackets) for these major outcome measures are given for Fruitflow and for control interventions. Significant differences from baseline measurements are indicated with individual *P* values at all instances. Significant differences from control treatments—placebo or positive control treatments—(*P* < 0.05) are indicated by * where applicable

## Size and variance of the acute antiplatelet effect

**Fig. 5:**
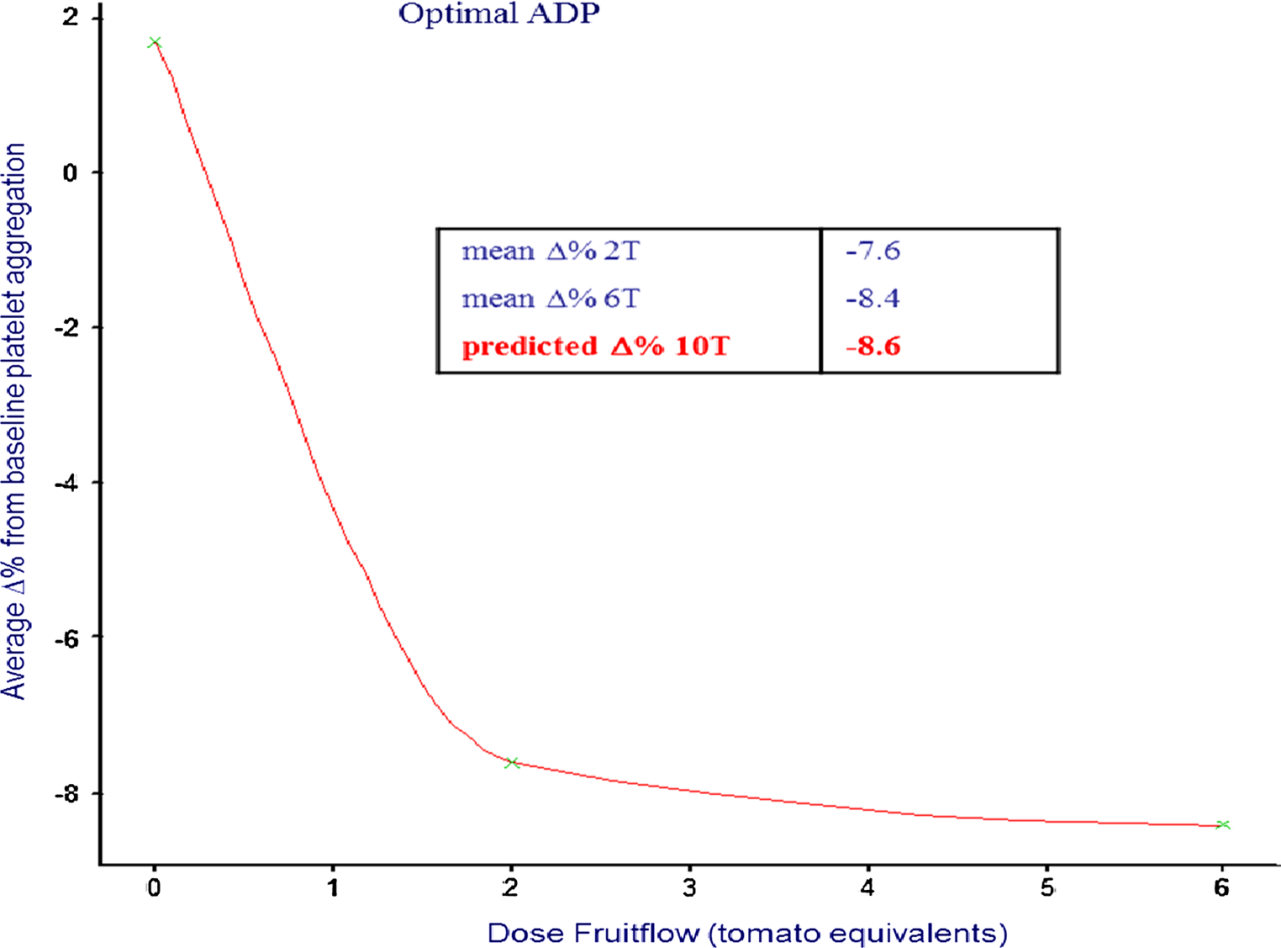
Dose–response curve at* t* = 3 h after ingestion of Fruitflow^®^ 1 at different dosage levels, in 23 subjects (Study #2, Table 5). Dose 2 tomatoes represents a single dose of Fruitflow^®^ 1 corresponding to 65 mg tAF components, while dose 6T represents a three doses corresponding to 195 mg tAF components. Data shown for dose 8 tomatoes (corresponding to 260 mg tAF components) are extrapolated from the curve generated

The range of acute antiplatelet effects observed in all studies undertaken is shown in Table 5. On average, these studies have shown an inhibition of the platelet response to ADP agonist of approximately 17–25 % and an inhibition of the response to collagen of approximately 10–18 %. Arachidonic acid-induced platelet aggregation and thrombin receptor-activating peptide (TRAP)-induced platelet aggregation have also been shown to fall after Fruitflow^®^ administration. A study in which Fruitflow^®^ 1 was administered to 93 healthy men and women (Table 5, #2) [16] showed that some variability in response may occur, with men responding more than women, and subjects with higher risk factors for CVD responding more highly than others. A dose–response was established in studies administering different amounts of Fruitflow^®^ 1 (Table 5, #1); the shape of the dose–response curve is shown in Fig. 5. This dose–response work established that a dose of Fruitflow^®^ equivalent to 65 mg tAF or approximately 3 average bowls of tinned tomato soup already caused close to the maximum level of platelet inhibition achievable by this extract and that no significant gain would be obtained in an acute setting from increasing the dose.

## Effects of ingredient format and food matrix on the observation of antiplatelet effects

Two studies have been undertaken focusing on the two different ingredient formats, Fruitflow^®^ 1 and Fruitflow^®^ 2, and the likelihood of matrix effects altering the acute antiplatelet response after consumption (Table 5, #4, #6). These studies, which involved 54 subjects, showed firstly that it was not possible to distinguish between the antiplatelet effects observed for the Fruitflow^®^ 1 and Fruitflow^®^ 2 ingredients, when consumed in doses of 3 g and 150 mg, respectively (Table 5, #4). No difference between the two ingredient formats could be detected three hours after consumption. Secondly, the studies showed that incorporation of WSTC into water-based drinks, yoghurts and capsules was all viable means of inducing an antiplatelet effect three hours after consumption (Table 5, #6). The antiplatelet effects observed in all matrices were similar to those seen in previous studies (Table 5).

### Effects of chronic consumption

Once the time of onset, persistence and size of effect related to a single dose had been established, studies examining the effects of continuous (i.e. daily) consumption of Fruitflow^®^ were undertaken. These studies showed that the size of the antiplatelet effect observed after consuming a single dose of Fruitflow^®^ daily for two or for four weeks was not significantly different from the size of effect observed after a single dose, that is, the observed effects were not cumulative (Table 5, #3). Suppression of platelet function achieved through chronic consumption was continuous—measurements of platelet function were taken in fasted subjects in the morning, approximately 24 h after consumption of their last Fruitflow^®^ dose, and suppression of original baseline platelet function was observed after two and four weeks.

## Safety considerations

Compounds found in Fruitflow^®^ have been shown to affect many aspects of platelet function, including (via effects on TF immobilization and signalling) thrombin generation. Therefore, during all human intervention studies, care was taken to incorporate specific safety-focused measures to examine whether any effects on the intrinsic or extrinsic clotting pathways could be detected alongside antiplatelet effects. An antiplatelet which also affected blood coagulation pathways could raise safety concerns. However, in all intervention studies undertaken, clotting time measurements showed no significant increases from baseline levels (Table 5). Fruitflow^®^ does not directly affect blood coagulation at any dose tested.

Even without affecting blood coagulation directly, many antiplatelet drugs, taken on a chronic basis, give rise to excessive platelet inhibition and are associated with internal bleeding. These potentially serious side effects mean that antiplatelet therapy, a fundamental aspect of CVD secondary prevention, is contraindicated for primary prevention as the benefit conferred (lowering risk of a first CVD event in relatively low risk groups) is outweighed by the increased risk of gastric or intracranial bleeding [49]. This judgement was recently revisited by the US FDA, in the context of increasing obesity and type 2 diabetes mellitus levels in relatively young populations, but was upheld [69]. The known side effects of existing antiplatelet drugs related to internal bleeding were clearly pertinent for consideration during Fruitflow^®^ development. However, Fruitflow^®^ differs fundamentally from antiplatelet drugs in the reversibility of its action. The widely used antiplatelet drugs have irreversible mechanisms of action. Over the course of ten days, approximately 90 % of the circulating platelet population can be irreversibly affected for the lifetime of those platelets. This level of platelet inhibition is then maintained by daily drug treatment. Conversely, the antiplatelet effects of Fruitflow^®^ are not irreversible, or cumulative, and can be overcome by increased agonist concentrations. This very significant difference in mode of action renders Fruitflow^®^ suitable for use by the general population as a dietary functional ingredient, while antiplatelet drugs cannot be used.

As Fruitflow^®^ is designed as a food ingredient, with potential for incorporation into a variety of food products, a specific study was undertaken to examine the likely effects of overconsumption (Table 5, #5). As the amount of Fruitflow^®^ in any one food product serving is low, equivalent to approximately three bowls of tinned tomato soup, and as dose–response studies had shown that increasing the dose significantly would not result in a much bigger acute effect on platelets, no significant dangers were anticipated. The results of the study confirmed this position; drinking 1L of a drink containing four daily doses of Fruitflow^®^ did not dangerously reduce platelet aggregability compared to baseline. In fact, the reduction in platelet functionality observed was rather less than that observed for a single serving of the same product. This underlines again the effects of food volume on observable systemic antiplatelet effects.

## Comparing the dietary antiplatelet Fruitflow^®^ with the antiplatelet drug aspirin

A study comparing a single dose of Fruitflow^®^ with 75 mg aspirin, either as a single dose or taken continuously for one week, was undertaken in order to benchmark the effects of a dietary antiplatelet (Table 5, #7). A comparison of the effects of Fruitflow^®^ and aspirin on the platelet proteome was first carried out, to examine similarities and differences in mechanisms of action. This comparison showed that aspirin and Fruitflow^®^ affect broadly similar proteomic pathways, with aspirin affecting the signalling pathways more strongly than Fruitflow^®^—assuming full metabolism of the entire ingested dose. Proteins affected by Fruitflow^®^ and aspirin are associated with platelet structure, platelet coagulation, platelet membrane trafficking and platelet secretion - actin-binding proteins, fibrinogen beta chain 5, Ras-related proteins, redox system proteins and HSP70s. Of the 26 proteins with altered expression after treatment, 11 were affected by both Fruitflow^®^ and aspirin, 14 by aspirin alone and one by Fruitflow^®^ alone. The single protein affected only by Fruitflow^®^ was identified as PDI, known to disrupt inside-out signalling as described earlier.

**Fig. 6:**
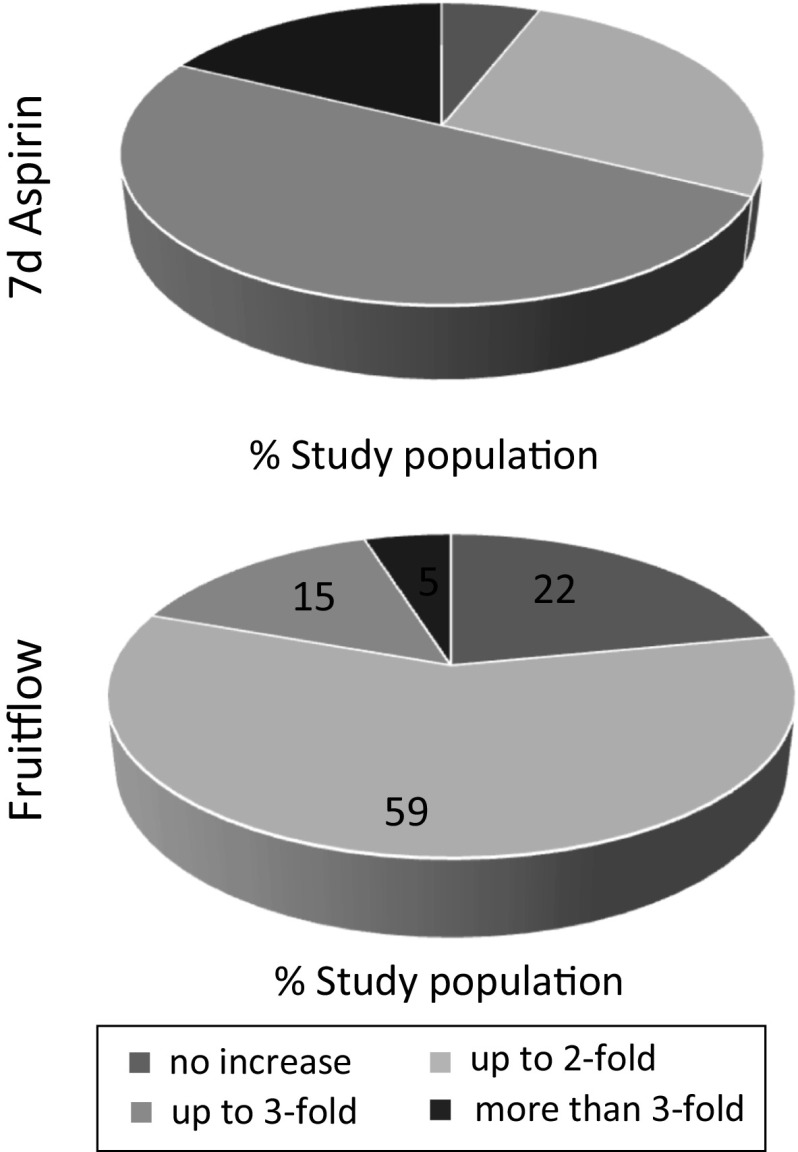
Increases in PFA-100 closure time by aspirin and Fruitflow^®^. Time to form a primary clot after treatment with either 7d aspirin (top) or Fruitflow^®^ (bottom) was determined. The proportions of the study population experiencing less than twofold, up to twofold and over threefold increases in time to form primary clot are shown, illustrating the heterogeneity of the responses observed

The intervention study which followed in 47 healthy subjects showed that the effects of a single dose of Fruitflow^®^ were similar, in terms of antiplatelet action, effects on thromboxane synthesis and time to form a primary haemostatic clot (PFA-100 closure time), to those of a single 75 mg dose of aspirin (Table 5, #7). When aspirin was taken daily for seven days, the associated increase in PFA-100 closure time was three times higher than that associated with a single aspirin dose. The cumulative antiplatelet effect of aspirin when taken daily is well known and reflects its irreversible disabling of platelet COX-1 and associated signalling. Fruitflow^®^’s effects are not cumulative in this way, as its effects do not irreversibly disable platelet signalling pathways. Thus, taking the results for the study population as a whole, daily aspirin supplementation may be viewed as approximately three times as efficacious as daily Fruitflow^®^ supplementation, due to the irreversibility of its action. This overall result seemed to echo the proteomic data, but further examination showed that it masked some interesting behaviour in study subgroups. The antiplatelet effects of aspirin in healthy subjects are extremely heterogeneous, with some subjects experiencing a very large increase in time to form a primary haemostatic clot, while others respond poorly (Fig. 6). Approximately 50 % of aspirin responders had a response to the drug which was lower than the average response for the treatment group, in terms of time to form a primary haemostatic clot. This group of subjects had a residually strong response to collagen after 7 days of aspirin treatment, and over one-third of the group responded better to Fruitflow^®^ supplementation than to 7-day aspirin supplementation. At the other end of the spectrum, for 18 % of the study population, taking aspirin for 7 days more than trebled the time to clot. This underlines the reasons behind the known internal bleeding risks associated with aspirin and its unsuitability for use in primary prevention. While the response to Fruitflow^®^, in terms of time to clot data, was also heterogeneous, it was markedly less so than the response to aspirin (Fig. 6). The majority of the subject group experienced increases in time to form a primary haemostatic clot of up to twofold, with less subjects at either extreme. It would appear that the proteomic predictions of stronger aspirin-led effects on platelet signalling may not be observed *ex vivo*, possibly due to wide variability in the extent of aspirin metabolism, but also possibly due to differences in the relative importance of platelet collagen signalling pathways between individuals. Fruitflow^®^, with its wider range of antiplatelet compounds, may have a less variable metabolism and thus achieve its more moderate effects more widely. These more moderate effects, which can be related to the reversibility of the antiplatelet action of Fruitflow^®^ rather than its mode of action per se, render it a possible option for use in primary prevention of CVD, in contrast to aspirin at any dosage. However, outcomes-based studies on dietary supplements such as Fruitflow^®^ are needed before their true potential can be properly assessed.

## EFSA approval of Fruitflow^®^ and its commercialization in different countries

In 2006, the European Union (EU) adopted a regulation on the use of nutrition and health claims for foods which lays down harmonized EU-wide rules for the use of health or nutritional claims on foodstuffs (Regulation (EC) No 1924/2006). One of the key objectives of this regulation is to ensure that any claim made on a food label in the EU is clear and substantiated by scientific evidence. Different categories of claim are defined. Health claims are defined as pertaining to relationships between food and health either with regard to a function of the body (Article 13 claims), or with regard to reducing a risk factor for a disease (Article 14a claims), or with regard to children’s development (Article 14b claims). Nutrient claims are defined as pertaining to foods with particular nutritional properties with regard to either the energy, or the nutrients, they provide. Allowed nutrient claims are clearly defined within the regulation. However, companies wishing to associate their food or ingredient with a health claim must submit a dossier in support of the desired claim, which is then assessed by the EFSA Panel on Dietetic Products, Nutrition and Allergies. Key to the dossier is the inclusion of human intervention studies showing evidence of the health benefit claimed, for the food/ingredient of interest. More than 2,200 unique claims have been submitted for assessment to date, of which over 95 % have been Article 13 claims, with an overall approval rate of close to 10 %. The first Article 13 claim based on newly developed evidence or proprietary data [a special category under Article 13(5)] to be achieved, in December 2009, was for Fruitflow^®^, when the EU Commission authorized the health claim “water-soluble tomato concentrate (WSTC) I and II helps maintain normal platelet aggregation, which contributes to healthy blood flow”. The authorized claim was based on the eight human studies (seven proprietary), and seven non-human studies (three proprietary), conducted with Fruitflow^®^, which is given in Table 5.

Thus, Fruitflow^®^ is now authorized by EFSA for daily consumption, with the conditions that 3 g Fruitflow^®^ 1 or 150 mg Fruitflow^®^ 2 must be included in either foods (e.g. fruit juices, flavoured drinks or yoghurt drinks with a total volume up to 250 mL), or in powdered, tabletted or encapsulated food supplements (to be taken with up to 250 mL water).

## New functionalities of Fruitflow^®^: Anti-inflammatory and blood pressure-lowering effects

Atherosclerosis is considered as a chronic inflammatory disease of the arterial vessel wall and is a major cause of mortality and morbidity in the world [70–72]. Inflammation plays a critical role in the initiation and progression of atherosclerosis; it involves the recruitment of inflammatory cells from the circulation and their transmigration through the endothelial wall, resulting in vascular damage, narrowing and hardening of the arteries [71]. The intake of tomatoes and tomato products has been associated with a decreased risk of chronic inflammatory diseases such as atherosclerosis and is linked with a healthy Mediterranean diet [1, 33, 73]. Moreover, several in vitro animal and human studies have shown that tomato concentrates and phytochemicals from tomato have anti-inflammatory activities and may reduce the risk of CVD [73–78].

**Fig. 7:**
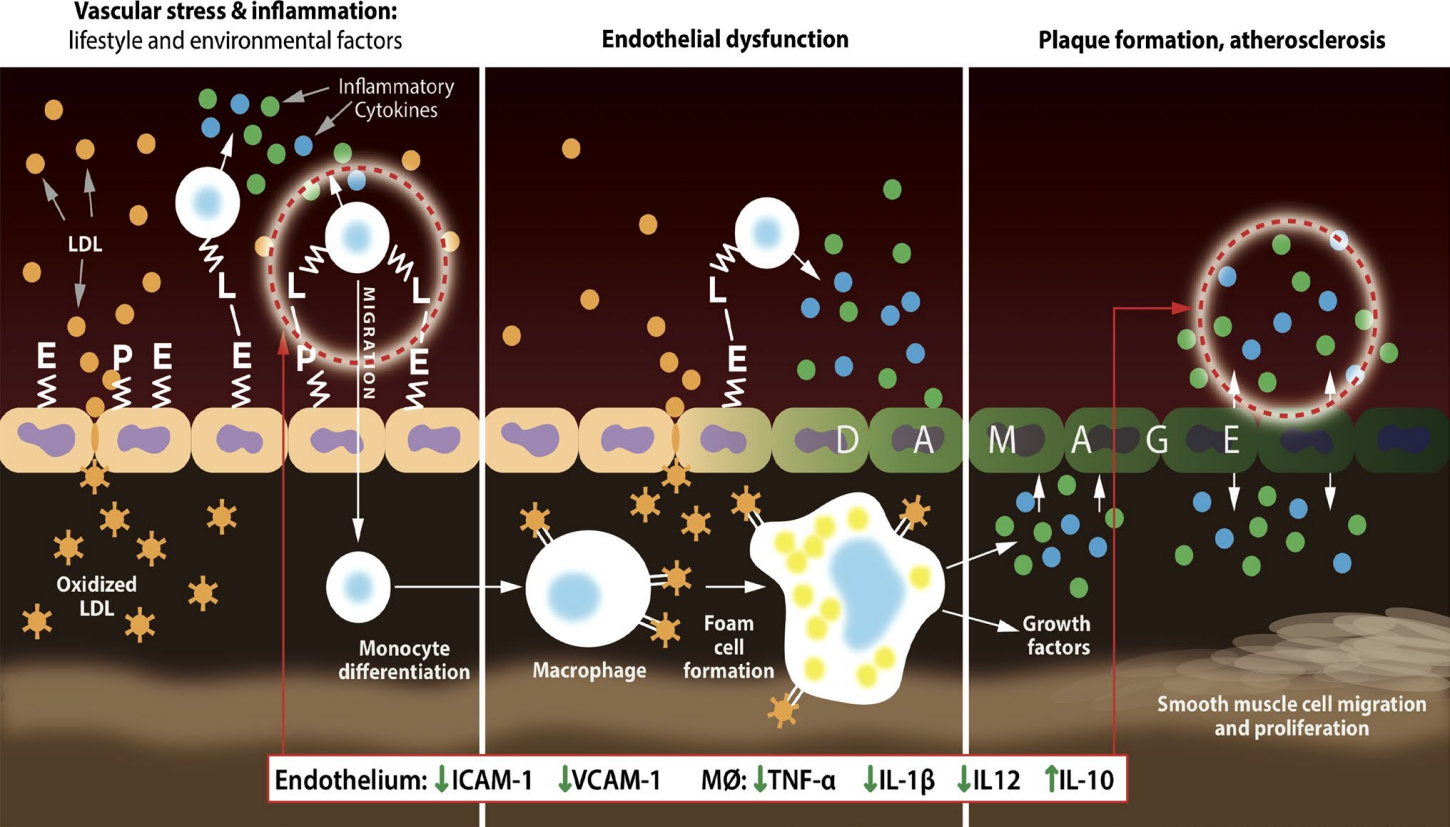
Fruitflow^®^ inhibits the activation of macrophage and endothelial cells via different mechanisms. The endothelium reacts to inflammatory stimuli derived from activation of cytokines such as NF-κB, and the up-regulation of adhesion molecules (ICAM, VCAM), which play a role in leucocyte adhesion and transmigration across the endothelial layer. NF-κB is a key transcription factor which induces the release of pro-inflammatory mediators (cytokines, chemokines and adhesion molecules) that further promote vascular inflammation leading to the initiation and development of atherosclerosis. This Fruitflow^®^ inhibits the activation of macrophage and endothelial cells, which could help to reduce leucocyte recruitment and promote a reduction in tissue inflammation

Activation of the inflammatory pathways in macrophages plays a critical role in the initiation and progression of endothelial dysfunction which eventually results in atherosclerosis. The influence of Fruitflow^®^ on the inflammatory response of macrophages and on endothelial dysfunction in human umbilical vein endothelial cells (HUVEC) has recently been investigated [18]. In this study, Fruitflow^®^ was able to regulate the production of cytokines and interleukins in LPS-stimulated macrophages. It decreased the production of pro-inflammatory cytokines (TNF-α, IL-1β and IL-12), while the production of the anti-inflammatory IL-10 was increased. Consequently, Fruitflow^®^ reduced the production of inflammatory mediators related to chronic inflammation. Nuclear factor-κB (NF-κB) is an important pro-inflammatory transcription factor, and its activation is involved in the pathology of vascular inflammatory diseases. Gene expression analysis indicated that Fruitflow^®^ reduced the expression levels of NF-κB, suggesting that Fruitflow^®^ regulated the gene expression of inflammatory mediators via the NF-κB pathway. The data are in agreement with the recent publication from Navarrete et al. [79] which investigated the effects of an aqueous extract of tomato (produced in a similar manner to the laboratory-scale aqueous extracts described here) on the expression of pro-inflammatory cytokine in LPS-activated monocyte-derived THP-1 macrophages. The Navarrete aqueous tomato extract was shown to inhibit the expression of cytokines (TNF-α, IL-1β) and the activation of NF-κB in the LPS-stimulated macrophages. Intercellular adhesion molecule-1 (ICAM-1) and vascular cell adhesion molecule-1 (VCAM-1) are two critical adhesion molecules that are expressed on endothelial cells and mediate adhesion of leucocytes and their interactions with inflamed endothelial cells leading to endothelium damage [80]. Activation of the endothelial cells and the production of inflammatory mediators by atherosclerotic lesions results in the up-regulation of the adhesion molecules (ICAM-1, VCAM-1). Thus, ICAM-1 and VCAM-1 are considered as early markers of endothelial dysfunction and atherosclerosis. In the Schwager et al study (2016), Fruitflow^®^ significantly decreased the production and the gene expression of ICAM-1 and VCAM-1 in activated HUVEC. This suggests that Fruitflow^®^ may be capable of altering some of the pathologies typically seen in endothelial dysfunction. Tomato is a rich source of lipophilic and hydrophilic phytochemicals which could influence the inflammatory response [73]. Fruitflow^®^ is standardized using three “natural” constituents: adenosine, chlorogenic acid and rutin, which are representative of the three main groups of active constituents and have been shown to inhibit both platelet aggregation and inflammatory pathways. Adenosine has been suggested to play a role in immune and inflammatory processes and to improve various inflammatory diseases [81–84]. Chlorogenic acid is a polyphenol with anti-inflammatory activity, which inhibits the LPS-triggered activation of NF-κB and the associated inflammatory response [85, 86]. In macrophages, rutin suppressed oxidative stress and modulated the gene expression of mediators involved in chronic inflammation [87, 88] [89]. Collectively, the data indicate that compounds contained in Fruitflow^®^ have the capacity to modulate signalling pathways which alter vascular function, the development of atherosclerotic lesions and consequently the risk of CVD by various mechanisms including anti-inflammatory effects. The extent to which such pathways can be affected in humans after ingestion of Fruitflow^®^ is not yet known and could form an interesting topic for future studies. Figure 7 summarizes the effects of Fruitflow^®^ on the endothelial response to inflammatory stimuli via different mechanisms such as activation of NF-κB and the up-regulation of adhesion molecules.

Hypertension is another very important risk factor for CVD including coronary heart disease, stroke, congestive heart failure and peripheral vascular disease [90]. Clinically, hypertension is characterized by abnormally high blood pressure with the systolic reading greater than 140 mmHg and diastolic reading greater than 90 mmHg. Lowering blood pressure has been linked to 35 %–40 % reductions in the incidence of stroke, 20 %–25 % in myocardial infarction and up to 50 % in heart failure, making hypertension a strong target for therapeutic intervention. Because dietary changes are a first-line intervention for hypertension, food products themselves are increasingly under consideration for the specific effects they may have on blood pressure. In recent years, there has been considerable interest in the potential for using natural food components as functional foods to treat hypertension, especially for people with borderline to mild high blood pressure that does not warrant the prescription of antihypertensive drugs. Increasingly, nutraceuticals and food products have been investigated for their effects on hypertension. Polyphenols inhibit and down-regulate expression of angiotensin-converting enzyme (ACE) and renin. In addition, polyphenols have also been associated with the formation of endothelial nitric oxide leading to vasodilation and lowering of blood pressure. The renin–angiotensin system is a powerful mechanism for controlling blood pressure [91, 92]. In hypertensive patients with elevated plasma rennin–angiotensin activity, a fivefold increased incidence of myocardial infarction was demonstrated [93]. ACE (EC 3.4.15.1, dipeptidyl carboxypeptidase) is a glycoprotein peptidyldipeptide hydrolase that cleaves histidyl leucine dipeptide from angiotensin I forming the potent vasoconstrictor angiotensin II. Studies demonstrated that ACE inhibitors (ACEIs) significantly reduced the morbidity and mortality in patients with myocardial infarction and the incidence of ischaemic events in patients with CVD, even in the absence of their blood pressure-lowering effects [94–96]. The therapeutic administration of certain ACEIs has also been associated with positive health effects beyond the regulation of blood pressure [97]. Polyphenols inhibit and down-regulate expression of ACE and renin [98]. Tomato juice was earlier shown to lower blood pressure in human volunteers, and this effect was thought to be associated with its high antioxidant content [99, 100]. We therefore tested whether Fruitflow^®^ inhibited human serum ACE activity and rabbit lung ACE activity [17]. The IC_50_ value of Fruitflow^®^ for ACE inhibition in serum was 1.91 ± 0.24 mg/ml (0.038 μg catechin equivalent(CE)/ml), whereas for captopril, the value was 0.56 ± 0.08 μg/ml. Fruitflow^®^ also inhibited rabbit lung ACE activity in a dose-dependent manner. Orange or banana extract had no such anti-ACE activity. Flavonoid-rich plant extracts have been demonstrated as natural competitive ACEIs where the ACE activity is identified as a critical factor in regulating high blood pressure. These compounds are known to be inhibitors of cyclic nucleotide phosphodiesterase and TxA2 synthesis, two main determining factors in human blood platelet activation/aggregation processes. Consequently, it is possible that the consumption of these bioactive components of Fruitflow^®^ might reduce more than one CVD risk factor, such as platelet hyperactivity and hypertension. As yet, these data are based on in vitro experiments, and it is clear that further work is required to provide clear information on the metabolic fate of these compounds, and the dosage required for physiological benefits. While a functional food approach offers interesting possibilities for cardio-protection, the requirement for long-term randomized controlled trials remains. Advances in the knowledge of both platelet biology and the mechanism of action of Fruitflow^®^ bioactive compounds will provide new avenues to develop dietary strategies aimed at promoting cardiovascular health.

## Fruitflow^®^ and other platelet inhibiting food ingredients

Nutritional modification of cellular functions by dietary lipids and other nutritive and non-nutritive factors offers an attractive avenue to correct, modify or prevent many pathophysiological processes including platelet hyperactivity [101]. The mediation of such effects is thought to be primarily achieved through alterations of cell membrane composition and other endogenous lipid stores, with a consequent reduction in AA-derived eicosanoid production and modification of the functional activity of various receptors on platelet membranes [101]. Eicosapentaenoic acid, 20:5n-3 (EPA) and DHA substitute biologically less potent eicosanoids, while the non-nutritive compounds act to reduce all eicosanoid formation specially TxA_2_, an important platelet-aggregating agent. Numerous epidemiological studies and clinical trials have reported the health benefits of different omega 3 polyunsaturated fatty acids (PUFAs), including a lower risk of coronary heart diseases [102]. There are several reviews available on the effects of alpha-linolenic (ALA), EPA and docosahexaenoic, 22:6n-3 (DHA) acids on some risk factors associated with atherothrombosis, including platelet activation, plasma lipid concentrations and oxidative modification of LDL [102, 103]. The role of platelets in haemostasis and thrombosis has been known for a long time and is well defined, but more recently a new concept has emerged stating that platelets play a central role in the atherothrombotic process [9, 10]. Dyerberg and Bang showed that Inuits had attenuated platelet reactivity [104] as they consumed omega 3 fatty acids. Platelet aggregation response was diminished with omega 3 fatty acids consumption [105–107]. A meta-analysis conducted by Gao et al. [108] has demonstrated that omega 3 PUFAs are associated with a significant reduction in platelet aggregation. In addition, low intake of EPA may reduce platelet aggregation, without changing the fatty acid platelet composition [109, 110]. In the same way, following supplementation with DHA [111] platelet function was reduced and a retroconversion of DHA into DPA and EPA was evidenced. More recently, a dose–response study with middle-aged healthy volunteers ingesting increasing amounts of DHA indicated that platelet reactivity was decreased after 400 and 800 mg DHA/day [112]. It is difficult to attribute precisely the effects to each fatty acid, and their beneficial and complementary effects could well be linked to formation or diminution of specific eicosanoids and/or docosanoids.

Combinations of antiplatelet drugs are known to have different physiological effects than single agents. Taking a similar strategy from a functional food perspective, a combination of tomato extract with antiplatelet activity [13] and n-3 fatty acids was investigated for effects on platelet aggregation in vitro. The combination of tomato extract and omega 3 fatty acids inhibited in vitro platelet aggregation to a greater extent than either alone, and this inhibition was correlated with intracellular platelet cAMP levels [113]. Such data indicate that combinations of Fruitflow^®^ and omega 3 fatty acids—and potentially other non-nutritive compounds such as resveratrol and other polyphenols—may be effective in improving platelet function. Further human intervention studies should be conducted in order to determine the scale of benefits which may arise following consumption of Fruitflow^®^ combined with other bioactive substances.

## Conclusions

This review provides information that substantiates the cardio-protective claims of Fruitflow^®^. Normal platelet activity is the key for the maintenance of haemostasis and normal blood flow. Hyperactive platelets interact with vessel walls by shedding macro-particles, secreting several adhesive growth factors, and inflammatory agents interrupt the blood flow and produce a pro-thrombotic state in people with obesity, diabetes, a sedentary lifestyle or hypertension, and in people who smoke. In general, the molecular events underpinning these processes are broadly similar. It has long been known that disturbances in blood flow, changes in platelet reactivity and enhanced coagulation reactions facilitate pathological thrombus formation, and the maintenance of normal platelet activity is critical to overall haemostasis. Fruitflow^®^ developed from tomato containing bioavailable cardio-protective compounds can be of benefit to the people who are vulnerable to develop CVD. The outlined data suggest that Fruitflow^®^ may be useful in the primary prevention of CVD. An array of extensive basic, mechanistic, compositional and several human trials are testimony to its cardio-protective benefits.

## Abbreviations

CVD: Cardiovascular disease
tAF: Total active fraction
ACE: Angiotensin-converting enzyme
EPA: Eicosapentaenoic acid, 20:5n-3
TRAP: Thrombin receptor-activating peptide
DHA: Docosahexaenoic, 22:6n-3
WSTC: Water-soluble tomato concentrate
EFSA: European Food Safety Authority
PDI: Protein disulphide isomerase
TF: Tissue factor
PRP: Platelet-rich plasma
VSMC: Vascular smooth muscle cells
TxA_2_: Thromboxane A_2_
